# Circulating Tumor DNA Testing in Curatively Resected Colorectal Cancer and Salvage Resection

**DOI:** 10.1001/jamanetworkopen.2024.52661

**Published:** 2024-12-27

**Authors:** Jingran Ji, Chongkai Wang, Ajay Goel, Kurt Melstrom, Yasmin Zerhouni, Lily Lai, Laleh Melstrom, Mustafa Raoof, Yuman Fong, Andreas Kaiser, Marwan Fakih

**Affiliations:** 1 Department of Medical Oncology and Therapeutics Research, City of Hope Comprehensive Cancer Center, Duarte, California; 2 Department of Molecular Diagnostics and Experimental Therapeutics, City of Hope Comprehensive Cancer Center, Duarte, California; 3 Department of Surgery, City of Hope Comprehensive Cancer Center, Duarte, California

## Abstract

**Importance:**

Serial circulating tumor DNA (ctDNA) has emerged as a routine surveillance strategy for patients with resected colorectal cancer, but how serial ctDNA monitoring is associated with potential curative outcomes has not been formally assessed.

**Objective:**

To examine whether there is a benefit of adding serial ctDNA assays to standard-of-care imaging surveillance for potential curative outcomes in patients with resected colorectal cancer.

**Design, Setting, and Participants:**

In this single-center (City of Hope Comprehensive Cancer Center, Duarte, California), retrospective, case cohort study, patients with stage II to IV colorectal cancer underwent curative resection and were monitored with serial ctDNA assay and National Cancer Center Network (NCCN)–guided imaging surveillance from September 20, 2019, to April 3, 2024. The median duration of follow-up was 26 months (range, 2-54 months).

**Interventions:**

Serial ctDNA assays were performed every 3 months for 2 years and every 6 months for the 3 following years in conjunction with NCCN-guided radiographic surveillance.

**Main Outcomes and Measures:**

The primary outcome was the proportion of patients with clinical benefit from ctDNA testing, defined as the proportion of patients with a newly positive ctDNA assay and negative scheduled imaging (most recent or concurrent) that subsequently led to early imaging confirmation of recurrence, followed by curative-intent intervention with no evidence of recurrence at the time of data cutoff. Recurrence was categorized by ctDNA recurrence, radiographic recurrence, or concurrent ctDNA and imaging recurrence. Salvage resections and associated durable remissions were described within each of the 3 categories. Descriptive statistics were used to characterize the patient population.

**Results:**

In total, 184 patients (median age, 59 years [range, 32-88 years]; 97 female [52.7%]) were included in this study, and 129 (70.1%) had stage II to III disease. Forty-five patients (24.5%) had ctDNA or imaging-confirmed recurrence. Of these 45 patients, 14 had radiographic recurrence with negative ctDNA, and 11 had concurrent ctDNA and imaging recurrence. Twenty of 45 patients had ctDNA positivity with negative imaging at first ctDNA positivity; 6 had reflex imaging that was positive for recurrence, and 14 continued with serial imaging and ctDNA monitoring. Ten of 14 patients had subsequent recurrent disease, 3 patients had a spontaneous clearance of ctDNA, and 1 patient remained imaging negative 7 months after positive ctDNA, after which she was lost to follow-up. Altogether, 11 of 20 patients with ctDNA recurrence without initial concurrent imaging recurrence had subsequent metastasectomy, and only 3 were disease-free at the cutoff date in April 2024, representing 1.6% of the surveilled population.

**Conclusions and Relevance:**

In this cohort study of patients with stage II to IV colorectal cancer who underwent curative-intent resection, the addition of serial tumor-informed ctDNA assay to the standard NCCN-recommended surveillance had limited clinical benefits. Additional prospective research is needed to clarify the value of ctDNA testing in the surveillance setting.

## Key Points

**Question:**

Is surveillance with a serial circulating tumor DNA (ctDNA) assay associated with improved chances of curative interventions in patients with resected colorectal cancer with disease recurrence?

**Findings:**

In this cohort study of 184 patients with resected stage II to IV colorectal cancer, incorporating serial ctDNA assays along with National Cancer Center Network–guided radiographic surveillance led to curative surgical intervention in 1.6% of patients.

**Meaning:**

These findings suggest that the addition of serial ctDNA assays to National Cancer Center Network–recommended imaging surveillance has limited clinical benefits; more research is needed to determine whether the addition of ctDNA assays to surveillance improves relevant clinical outcomes over the standard of care and what the optimal frequency of testing may be.

## Teaser

This cohort study examines the potential curative clinical outcomes of adding serial circulating tumor DNA assays to standard-of-care imaging surveillance in patients with resected colorectal cancer (CRC).

## Introduction

Despite advances in surgical techniques and adjuvant therapy for patients with resected colorectal cancer (CRC), a substantial number of patients continue to have disease recurrence with poor outcomes.^1,2,3,4^ As a result, the surveillance strategy for patients with resected locoregional and metastatic CRC remains an important area of study. Although there is some continued disagreement in the existing literature regarding the optimal surveillance schedule,^5,6,7,8,9,10^ the National Comprehensive Cancer Network (NCCN) recommendation for imaging surveillance for patients with high-risk stage II and stage III disease is computed tomography (CT) of the chest, abdomen, and pelvis every 6 to 12 months for 2 years, then yearly imaging for 3 years.^11^ For resected stage IV disease, the recommendation is CT imaging every 3 to 6 months for 2 years, then every 6 to 12 months for 3 years. The European Society of Medical Oncology recommends a similar surveillance strategy.^12^

With the recent clinical availability of circulating tumor DNA (ctDNA) assays, there has been substantial interest in the benefit of incorporating ctDNA assays into the surveillance strategy for patients with stage II to III and resected stage IV CRC. One such assay that has received considerable attention is Signatera (Natera), a personalized next-generation sequencing assay for ctDNA detection that is informed by patient-specific tumor DNA. Studies examining ctDNA after curative intent resection demonstrated that ctDNA positivity is associated with an increased risk for clinical relapse, even after completion of adjuvant chemotherapy.^13,14,15,16^ Preliminary results from more recent prospective trials^17,18,19,20^ have further confirmed that ctDNA positivity both before and after resection is a robust prognostic indicator of disease recurrence and time to recurrence. Furthermore, sustained clearance of ctDNA is associated with high rates of disease-free survival, and ctDNA positivity after surgery was associated with benefit from adjuvant chemotherapy.^17,18^

Yet, there remain limited data on how the use of serial ctDNA assays in the surveillance setting is associated with clinically meaningful outcomes in patients with resected CRC. The initial observational study that led to the enthusiasm behind the implementation of the ctDNA assay into longitudinal surveillance showed that serial evaluation with ctDNA assays every 3 months for 36 months after definitive treatment for stage II to III CRC led to the detection of recurrent disease at a median of 8.7 months before imaging findings.^21^ However, the imaging surveillance used in that previous study was done at years 1 and 3 after definitive treatment, which is far less frequent than the current NCCN and European Society of Medical Oncology guidelines. We have previously evaluated the sensitivity of ctDNA in detecting a confirmed recurrence of disease compared with imaging alone using an NCCN-compliant imaging schedule. We found that the sensitivity of the 2 modalities was similar and that the time to detection of disease recurrence was the same.^22^ More recent data from the BESPOKE trial^17^ suggested that ctDNA may detect recurrence before imaging in a subset of patients, but the clinical outcomes of this early detection are not clear. Here, we describe a single institutional experience regarding the potential curative clinical outcomes with the addition of serial ctDNA assays to standard-of-care imaging surveillance, as recommended by NCCN guidelines.

## Methods

We performed a retrospective cohort study at a single academic center (City of Hope Comprehensive Cancer Center, Duarte, California) to assess the outcomes of adding serial ctDNA testing with a Clinical Laboratory Improvement Amendments–certified ctDNA assay on the potential for curative intervention at the time of recurrence among patients with resected stage II to IV CRC. This study was approved by the institutional review board at the City of Hope National Comprehensive Cancer Center. A waiver of informed consent was obtained, because the data were deidentified, in accordance with 45 CFR §46. The results reporting adheres to Strengthening the Reporting of Observational Studies in Epidemiology (STROBE) reporting guidelines.

All patients with CRC who had ctDNA testing as part of their surveillance schedule from September 20, 2019, to April 3, 2024, were identified. All patients included were monitored with serial ctDNA testing every 3 months for 2 years, then every 6 months for 3 years after curative-intent surgery. Surveillance CT scans were performed, per NCCN guidelines, every 6 months for 2 years and then every year for 3 years for patients with high-risk stage II and III disease. Imaging studies were performed every 3 to 4 months for 2 years and then every 6 months for 3 years for those with resected stage IV disease. For those with low-risk stage II disease, imaging was performed every year for 5 years. If a patient had a positive ctDNA assay prior to scheduled imaging, a reflex CT or positron emission tomography–CT scan was done and then repeated every 3 months as long as ctDNA remained positive or until confirmed recurrent disease.

Recurrent disease was defined as evidence of recurrence on imaging or ctDNA positivity. We divided the study population into 4 cohorts (Figure): (1) those with negative ctDNA assays and negative scheduled imaging (either concurrent or most recent imaging), (2) those with a positive ctDNA assay and negative scheduled imaging, (3) those with a negative ctDNA assay and positive scheduled imaging, and (4) those with a concurrently positive ctDNA assay and scheduled imaging. Patients with a positive ctDNA assay and negative scheduled imaging were further divided into those with reflex imaging (additional imaging triggered by ctDNA positivity) positive or negative for recurrence. All patients were evaluated for recurrence, metastasectomy, recurrence following metastasectomy, and ctDNA clearance in those without radiographic recurrence.

**Figure.**
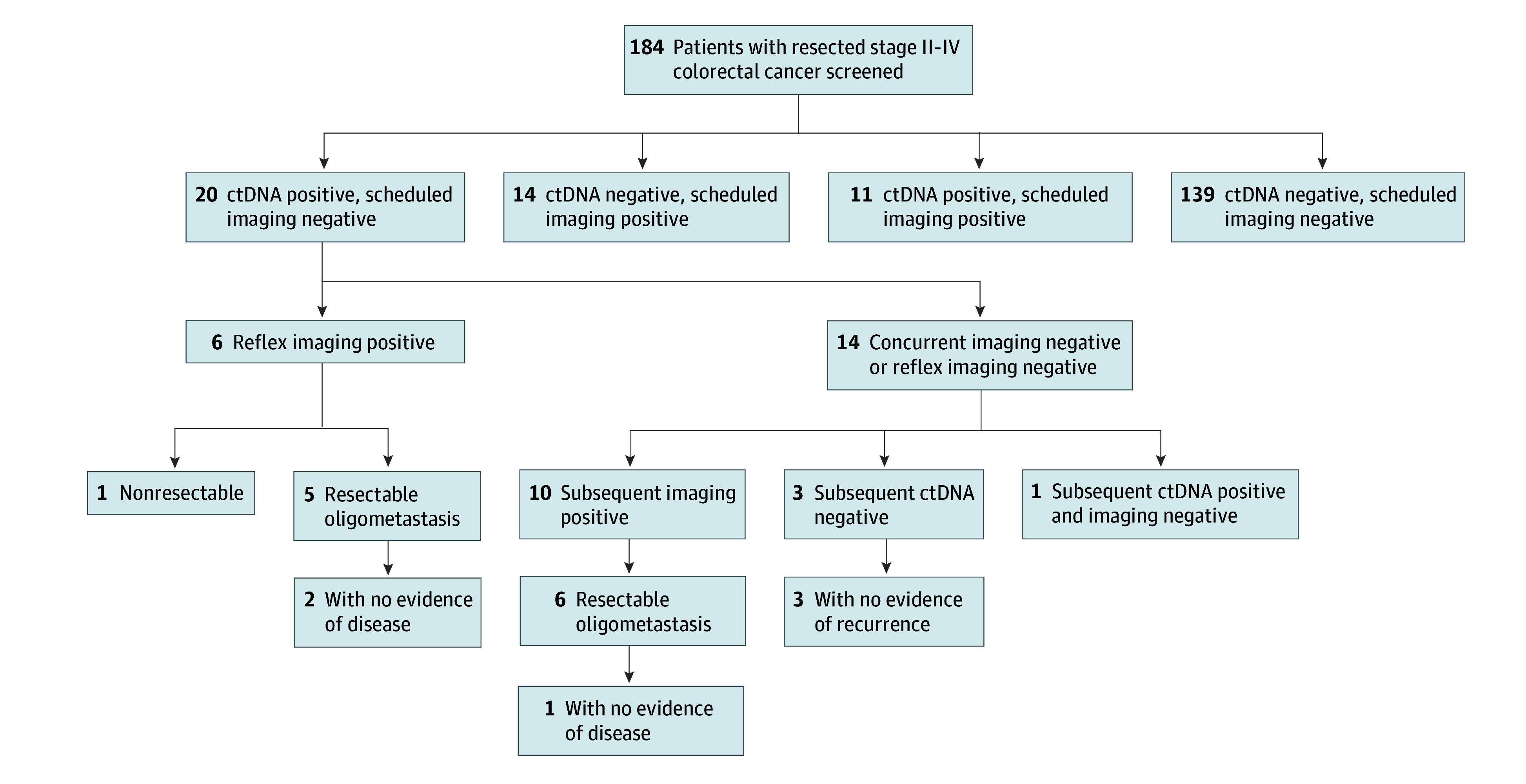
Recurrence Patterns During Surveillance With Circulating Tumor DNA (ctDNA) Assay and Imaging for Stage II to IV Colorectal Cancer

### Statistical Analysis

The number of patients included in this study was determined by the number of eligible patients who had resected CRC and underwent serial ctDNA testing during surveillance from 2019 to 2024. The primary outcome was the proportion of patients with clinical benefit from ctDNA testing, defined as the proportion of patients with a newly positive ctDNA assay and negative scheduled imaging (most recent or concurrent) that subsequently led to early imaging confirmation of recurrence, followed by curative-intent intervention with no evidence of recurrence at the time of data cutoff. The study cohort was evaluated as a whole and then separately for patients with stage II to III disease and those with resected stage IV disease. We used descriptive statistics to characterize the patient population. Excel software version 2410 (Microsoft) was used to manage the data.

## Results

### Patient Characteristics

A total of 184 patients (median age, 59 years [range, 32-88 years]; 97 female [52.7%]) with resected stage II to IV CRC underwent surveillance with serial ctDNA assays and were included in this study; 129 (70.1%) had stage II to III disease (Table 1). Surveillance was conducted for a median duration of 26 months (range, 2-54 months) at the time of data cutoff.

**Table 1.** Patient Demographics and Disease Characteristics

Characteristic	Patients, No. (%) (N = 184)
Age, median (range), y	59 (32-88)
Sex	
Male	87 (47.3)
Female	97 (52.7)
Cancer stage	
II-III	129 (70.1)
IV	55 (29.9)
Tumor location	
Left colon and rectum	129 (70.1)
Right colon and transverse	52 (28.3)
Unknown	3 (1.6)

### Recurrence During Surveillance

Of the 184 patients in this study, 45 (24.5%) had imaging-confirmed or ctDNA-confirmed recurrence of disease (Figure). Of the 45 patients who had recurrent disease, 11 had recurrence with concurrently positive ctDNA and imaging findings, 14 had recurrence by imaging with a negative ctDNA assay, and 20 had a positive ctDNA-recurrence with negative imaging studies (either prior or concurrent). As expected, the relative recurrence rate was higher among those with surveilled resected stage IV disease (24 of 45 patients [43.6%]) compared with those with resected stage II to III disease (21 of 129 patients [16.3%]) (Table 2).

**Table 2.** Recurrence Patterns During Surveillance With ctDNA Assay and Imaging for Stage II to III vs Resected Stage IV Disease

Surveillance cohort	Patients, No. (%)	
	Stage II-III (n = 129)	Stage IV (n = 55)
ctDNA positive, imaging negative	10 (7.8)	10 (18.2)
Reflex imaging positive	3 (2.3)	3 (5.5)
Nonresectable	0	1 (1.8)
Resectable oligometastasis	3 (2.3)	2 (3.6)
No evidence of disease	1 (0.8)	1 (1.8)
Concurrent imaging negative	7 (5.4)	7 (1.3)
Subsequent imaging positive	4 (2.3)	6 (1.3)
Resectable oligometastasis	1 (0.8)	5 (9.1)
No evidence of disease	1 (0.8)	0
Subsequent ctDNA negative	2 (1.6)	1 (1.8)
No evidence of disease	2 (1.6)	1 (1.8)
Subsequent ctDNA positive, imaging negative	1 (1.6)	0
ctDNA negative, imaging positive	8 (6.2)	6 (10.9)
ctDNA positive, imaging positive	3 (2.3)	8 (14.5)
ctDNA negative, imaging negative	108 (83.7)	31 (56.4)

Abbreviation: ctDNA, circulating tumor DNA.

### Clinical Benefit of ctDNA Assay in Surveillance

Among the 20 patients with ctDNA positivity and negative imaging, 14 had concurrently negative imaging, and 6 had early reflex imaging showing evidence of recurrent disease (Figure). This cohort included patients with both stage II to III and resected stage IV disease, and most had received some form of systemic therapy before definitive surgical resection (Table 3). The median time from surgery to ctDNA positivity was 11 months (range, 0-39 months), and the most common site of recurrence was in the liver (8 patients [40.0%]), followed by lungs (4 patients [20.0%]) and distant lymph nodes (3 patients [15.0%]). Ultimately, 5 of the 6 patients with early reflex imaging underwent salvage resection or ablative therapy, but only 2 remained without evidence of disease.

**Table 3.** Surveillance Details for Patients in the ctDNA-Positive, Imaging-Negative Cohort

Patient No.	Stage at diagnosis	Neoadjuvant or adjuvant chemotherapy	Time from surgery to ctDNA positive, mo	Time from ctDNA positive to imaging positive, mo	Site of recurrence	Resectable disease	No recurrence after resection
1	II	No	13	Imaging negative	NA	NA	NA
2	II	Yes	11	ctDNA cleared	NA	NA	NA
3	III	Yes	6	Reflex imaging positive	Liver	Yes	Yes
4	III	Yes	9	6	Lung and liver	No	No
5	III	Yes	39	12	Ovary	Yes	No
6	III	Yes	26	Reflex imaging positive	Lymph nodes	Yes	No
7	III	Yes	14	Reflex imaging positive	Liver	Yes	No
8	III	Yes	11	ctDNA cleared	NA	NA	NA
9	III	Yes	0	18	Retroperitoneum	No	No
10	III	Yes	18	18	Lymph nodes	No	No
11	IV	Yes	11	3	Lung	Yes	No
12	IV	Yes	4	3	Liver	Yes	No
13	IV	Yes	12	6	Peritoneum	Yes	No
14	IV	Yes	8	3	Liver	Yes	No
15	IV	Yes	15	3	Liver	Yes	No
16	IV	Yes	9	Reflex imaging positive	Lung	Yes	No
17	IV	Yes	30	6	Lung	No	No
18	IV	Yes	1	ctDNA cleared	NA	NA	NA
19	IV	Yes	1	Reflex imaging positive	Lymph nodes	No	No
20	IV	Yes	1	Reflex imaging positive	Liver	Yes	Yes

Abbreviations: ctDNA, circulating tumor DNA; NA, not applicable.

Of the 14 patients with concurrent negative imaging, 10 had subsequent imaging that eventually showed recurrent disease, and 6 of these patients underwent local therapy for oligometastatic disease. Among these 6 patients, 2 had recurrence in the liver, 2 in the lung, 1 in the ovary, and 1 in the retroperitoneum. Ultimately, only 1 patient in this cohort remained without evidence of disease as of the cutoff date. Of the remaining 4 patients who did not have subsequent imaging recurrence, 1 patient had continued ctDNA positivity on serial testing with persistently negative scans at the time of data cutoff. This patient had resected stage II disease and a first positive ctDNA assay 13 months after definitive surgery and was lost to follow-up after a negative scan 7 months later. Three patients spontaneously cleared their ctDNA and continued to be ctDNA, imaging negative 27, 24, and 23 months, respectively, after initial clearance. Altogether, ctDNA testing led to further reflex or intensive imaging with 11 patients undergoing surgical or ablative therapy, but only 3 of whom remained without disease recurrence as of the cutoff date, corresponding to a potential curative outcome in 1.6% of the entire screened population (3 of 184 patients).

### Patients With Imaging Recurrence and Negative ctDNA Assays

Fourteen patients (7.6%) from the overall cohort had a negative ctDNA assay at the time of imaging detected recurrence (Table 4 and eFigure, panel A, in Supplement 1). Of the 14 patients, most had stage II to III disease (10 patients [71.4%]), and the majority had lung-only recurrences (10 patients [71.4%]). One patient had a recurrence in the peritoneum, 1 in the abdominal wall, 1 in a pelvic lymph node, and 1 in the liver. The median time from surgery to recurrence was 14 months (range, 2-21 months). Of the patients identified with recurrence by imaging and without ctDNA positivity, 6 underwent salvage surgery or ablative procedures, and 4 remained without evidence of disease.

**Table 4.** Surveillance Details for Patients in the Circulating Tumor DNA–Negative and Imaging-Positive Cohort

Patient No.	Stage at diagnosis	Neoadjuvant or adjuvant chemotherapy	Time from surgery to imaging positive, mo	Site of recurrence
1	II	Yes	21	Abdominal wall
2	II	Yes	9	Lung
3	III	Yes	17	Lung
4	III	Yes	6	Lung
5	III	Yes	15	Pelvis or peritoneum
6	III	Yes	17	Lung
7	III	No	15	Lung
8	III	Yes	2	Pelvic lymph node
9	III	Yes	12	Lung
10	III	Yes	9	Lung
11	IV	Yes	8	Lung
12	IV	Yes	19	Lung
13	IV	Yes	16	Lung
14	IV	Yes	5	Liver

### Patients With Concurrent Imaging Recurrence and Positive ctDNA Assays

Eleven patients (6.0%) had both a positive ctDNA assay as well as positive imaging at the time of recurrence (eFigure, panel B, in Supplement 1). Of these 11 patients, most had stage IV disease (8 patients [72.7%]) and had recurrences in the liver (6 patients [54.5%]). The median time from surgery to recurrence was 7 months (range, 1-36 months); 6 patients underwent surgery or ablative procedures, with 3 remaining without evidence of disease.

### ctDNA Recurrence and Lag Time From Radiographic Recurrence

When analyzing the overall population, the median time to recurrence was similar for ctDNA and imaging. When focusing on the recurrent population by ctDNA testing (31 patients), the median time from recurrence by imaging from ctDNA positivity was less than 1 month, as 11 patients were concurrently recurrent by imaging and 6 were recurrent by reflex imaging. When limiting the analysis to the 20 patients who were ctDNA positive without concurrent positive imaging, the median time to evidence of disease recurrence was 3 months (range, 1-18 months).

## Discussion

In this single-institution, retrospective cohort study, we found that a surveillance strategy for stage II to III and resected stage IV CRC incorporating serial ctDNA assays provided potential curative clinical benefit for 1.6% of the study population. Although 20 patients had early ctDNA detection of recurrence prior to imaging findings, only 3 of 11 patients had oligometastatic recurrences that were addressed with curative intent and did not recur at the time of analysis. Furthermore, it is possible that these 3 patients would have been identified by subsequent NCCN-recommended imaging and still may have derived a curative outcome. In comparison, 12 of 25 patients identified by imaging as the first evidence of recurrence, with or without concurrent ctDNA positivity, underwent curative intent resections or ablative procedures, and 7 of them remained without evidence of disease as of the cutoff date.

These results contrast with the conclusions of the prospective Danish trial,^21^ which indicated that ctDNA allowed for much earlier detection of recurrence compared with imaging alone. Our findings suggest that there may be a more limited role for serial ctDNA testing in the surveillance setting when using a more frequent imaging schedule, as recommended by the current NCCN guidelines. More robust prospective studies, including cost-effectiveness studies, are needed to determine the value of serial ctDNA testing in the surveillance setting and to justify the incorporation of such assays in routine clinical practice. Recent preliminary data from the ongoing BESPOKE trial^17^ showed that ctDNA positivity may precede radiographic recurrence detection and increase targeted therapy for oligometastatic disease. However, the data are not yet mature, and the case-control design and the variable surveillance regimens in that study limit the interpretation of the findings.

Even if ctDNA assays can detect earlier recurrences compared with conventional imaging, limited data suggest that further intensifying surveillance and pushing for earlier detection of recurrence is directly correlated with improvements in survival. Studies done with older surveillance strategies do suggest that patients with disease recurrence picked up on routine testing are more likely to undergo successful curative-intent resection compared with those who are found to have recurrence after presenting with symptoms.^23,24,25^ Although a few studies have shown a modest survival benefit of more frequent follow-up during surveillance,^6,26^ several other randomized trials have failed to demonstrate that more-intensive surveillance regimens lead to a substantial survival benefit compared with less-intensive regimens.^7,9,10,27,28,29^ Indeed, these mixed results suggest that the clinical benefit of early detection of recurrence may plateau after a certain threshold, and further improvements to the timing of detection may not be worth the added cost of more frequent or, in the case of ctDNA assays, more novel testing.

In addition, the value of adding a blood-based marker for recurrence in combination with CT imaging has been extensively explored with the introduction of carcinoembryonic antigen (CEA) testing. CEA by itself is not highly sensitive or specific for CRC,^30^ but when measured serially in the setting of surveillance for recurrent CRC, it has been shown to detect recurrence several months before other modalities, including CT imaging alone.^31,32,33^ Although CEA has been shown to be potentially cost-effective,^34^ there remain no prospective data demonstrating that a clear survival benefit can be attributed to the addition of serial serum CEA measurements. Thus, it is difficult to justify the value of adding ctDNA assays, which are much more costly than CEA assays, to the current NCCN-recommended surveillance strategy of serial CEA testing and CT imaging. In addition, there is no definitive evidence to date that the implementation of systemic chemotherapy will have a curative outcome in patients with ctDNA recurrence and negative imaging in a surveillance setting. Hence, no treatment strategies can be recommended at this time for patients with ctDNA recurrent, imaging-negative CRC. Until novel treatment strategies are developed and validated for these patients who harbor microscopic disease, further intensifying surveillance is unlikely to translate into improvements in key clinical outcomes.

More recent data suggest that ctDNA assays may be more clinically useful when used instead as a biomarker of minimal residual disease after curative-intent surgery to guide systemic therapy. Previous studies and preliminary results from ongoing studies have established the prognostic value of ctDNA in this setting, where patients with persistent ctDNA positivity after surgery have substantially higher rates of relapse.^16,17,18,19,21^ Both the BESPOKE and GALAXY trials have also reported the benefit of adjuvant chemotherapy among those with ctDNA positivity, even after controlling for other relevant clinical risk factors such as stage.^17,18^ Furthermore, patients who are ctDNA negative may not benefit as much from adjuvant chemotherapy, and ctDNA may be used to guide the decision of adjuvant therapy in clinically lower-risk patients.^35^

### Limitations

There are several limitations to this study. First, the retrospective nature of this study limits the interpretation of our findings, and not all patients were followed for a prespecified period of time. Thus, only a short duration of the surveillance schedule was captured for some of the included patients, contributing to a relatively low prevalence of disease recurrence in this cohort. Second, the relatively small sample size may have led to an underestimation of the clinical impact of serial ctDNA assays. Third, our patients who underwent resection without recurrence have not completed 5-year follow-up; hence, we may have overestimated the clinical benefit. Fourth, because this was a single-institution experience, our surveillance strategy may vary from other institutions or community practices where practice patterns and resource limitations may result in less frequent imaging and increase the utility of serial ctDNA testing.

## Conclusions

The findings of this retrospective cohort study suggest that the addition of serial ctDNA assays to NCCN-recommended imaging surveillance of resected stage II to IV CRC may have limited clinical benefit. Future prospective trials are needed to evaluate whether the addition of ctDNA to surveillance improves relevant clinical outcomes, including patient-reported outcomes, over the standard of care and whether the frequency of testing is worth the cost and psychological impact on patients.

## Notes

Supplement 1. **eFigure.** Disease outcomes for patients with recurrence in the setting of positive imaging (A) without concurrently positive ctDNA and (B) with concurrently positive ctDNA

Supplement 2.
Data Sharing Statement
