# Error in Table 3

**DOI:** 10.1001/jamanetworkopen.2025.3590

**Published:** 2025-03-05

In the Original Investigation titled “Circulating Tumor DNA Testing in Curatively Resected Colorectal Cancer and Salvage Resection,”^1^ published December 27, 2024, there was an error in Table 3. The heading of the far-right column was incorrect and should have read, “No recurrence after resection.” This article has been corrected.^1^
