# Supplementary material for: Circulating Tumor DNA Testing in Curatively Resected Colorectal Cancer and Salvage Resection
Source: JAMA Netw Open. 2024 Dec 27;7(12):e2452661. doi: 10.1001/jamanetworkopen.2024.52661 (PMC11681374; doi:10.1001/jamanetworkopen.2024.52661)
Supplement: Supplement 1. — eFigure. Disease outcomes for patients with recurrence in the setting of positive imaging (A) without concurrently positive ctDNA and (B) with concurrently positive ctDNA [file jamanetwopen-e2452661-s001.pdf]

Supplemental Online Content

Ji J, Wang C, Goel A, et al. Circulating tumor DNA testing in curatively resected colorectal cancer and salvage resection. *JAMA Netw Open*. 2024;7(12):e2452661. doi:10.1001/jamanetworkopen.2024.52661

eFigure. Disease outcomes for patients with recurrence in the setting of positive imaging (A) without concurrently positive ctDNA and (B) with concurrently positive ctDNA

This supplemental material has been provided by the authors to give readers additional information about their work.

© 2024 Ji J et al. *JAMA Network Open*.

eFigure. Disease outcomes for patients with recurrence in the setting of positive imaging (A) without concurrently positive ctDNA and (B) with concurrently positive ctDNA

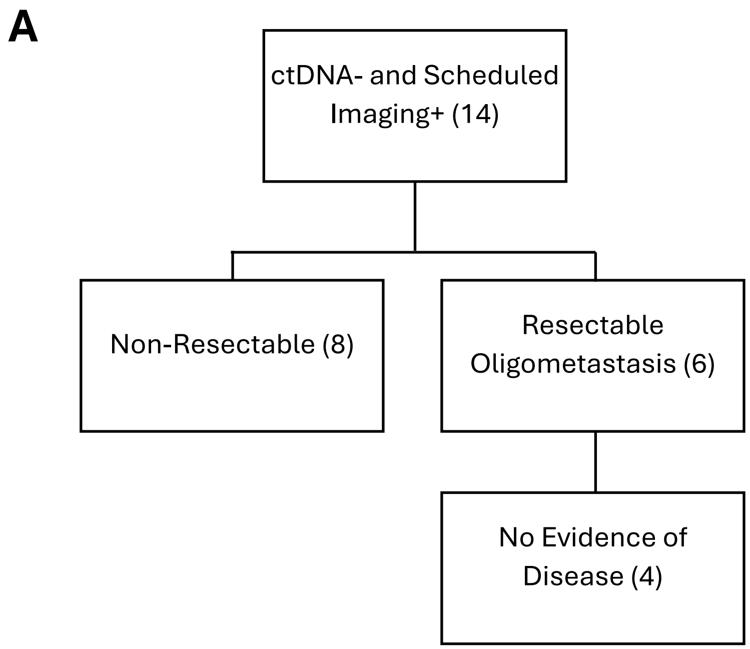

© 2024 Ji J et al. *JAMA Network Open*.
