# Supplementary material for: Circulating Tumor DNA Testing in Curatively Resected Colorectal Cancer and Salvage Resection
Source: JAMA Netw Open. 2024 Dec 27;7(12):e2452661. doi: 10.1001/jamanetworkopen.2024.52661 (PMC11681374; doi:10.1001/jamanetworkopen.2024.52661)
Supplement: Supplement 2. — Data Sharing Statement [file jamanetwopen-e2452661-s002.pdf]

Data Sharing Statement

Ji. Circulating Tumor DNA Testing in Curatively Resected Colorectal Cancer and Salvage Resection. *JAMA Netw Open*. Published December 27, 2024.
doi:10.1001/jamanetworkopen.2024.52661

Data
Data available: Yes
Data types: Deidentified participant data
How to access data: mfakih@coh.org
When available: With publication

Supporting Documents
Document types: None

Additional Information
Who can access the data: researchers whose proposed use of the data has been approved
Types of analyses: For any purpose
Mechanisms of data availability: With investigator support.
